# Recommendations for reporting tissue and circulating tumour (ct)DNA next-generation sequencing results in non-small cell lung cancer

**DOI:** 10.1038/s41416-024-02709-4

**Published:** 2024-05-15

**Authors:** Umberto Malapelle, Natasha Leighl, Alfredo Addeo, Dov Hershkovitz, Maximilian J. Hochmair, Ola Khorshid, Florian Länger, Filippo de Marinis, Nir Peled, Brandon S. Sheffield, Egbert F. Smit, Santiago Viteri, Jürgen Wolf, Filippo Venturini, Richard M. O’Hara Jr, Christian Rolfo

**Affiliations:** 1 Department of Public Health, University of Naples Federico II, Naples, Italy; 2 Department of Medical Oncology, Princess Margaret Cancer Centre, University Health Network, University of Toronto, Toronto, ON Canada; 3 Oncology Unit, Geneva University Hospital, Geneva, Switzerland; 4 Tel-Aviv Sourasky Medical Center, Tel-Aviv, Israel; 5 Department of Respiratory & Critical Care Medicine, Karl Landsteiner Institute of Lung Research & Pulmonary Oncology, Klinik Floridsdorf, Vienna, Austria; 6 National Cancer Institute, Cairo University, Cairo, Egypt; 7 Institute of Pathology, Hannover Medical School, Hannover, Germany; 8 Division of Thoracic Oncology, European Institute of Oncology, IRCCS, Milan, Italy; 9 Helmesely Cancer Center, Shaare Zedek Medical Center, Jerusalem, Israel; 10 Division of Advanced Diagnostics, William Osler Health System, Brampton, ON Canada; 11 Department of Pulmonary Diseases, Leiden University Medical Centre, Leiden, The Netherlands; 12 UOMI Cancer Center, Clínica Mi Tres Torres, Barcelona, Spain; 13 Lung Cancer Group Cologne, Center for Integrated Oncology, University Hospital of Cologne, Cologne, Germany; 14 Merck Serono S.p.A., Rome, Italy; 15 EMD Serono, Inc., Rockland, MA USA; 16 Center for Thoracic Oncology, Tisch Cancer Institute, Mount Sinai Medical System & Icahn School of Medicine, New York, NY USA

**Keywords:** Tumour biomarkers, Non-small-cell lung cancer

## Abstract

Non-small cell lung cancer is a heterogeneous disease and molecular characterisation plays an important role in its clinical management. Next-generation sequencing-based panel testing enables many molecular alterations to be interrogated simultaneously, allowing for comprehensive identification of actionable oncogenic drivers (and co-mutations) and appropriate matching of patients with targeted therapies. Despite consensus in international guidelines on the importance of broad molecular profiling, adoption of next-generation sequencing varies globally. One of the barriers to its successful implementation is a lack of accepted standards and guidelines specifically for the reporting and clinical annotation of next-generation sequencing results. Based on roundtable discussions between pathologists and oncologists, we provide best practice recommendations for the reporting of next-generation sequencing results in non-small cell lung cancer to facilitate its use and enable easy interpretation for physicians. These are intended to complement existing guidelines related to the use of next-generation sequencing (solid and liquid). Here, we discuss next-generation sequencing workflows, the structure of next-generation sequencing reports, and our recommendations for best practice thereof. The aim of these recommendations and considerations is ultimately to ensure that reports are fully interpretable, and that the most appropriate treatment options are selected based on robust molecular profiles in well-defined reports.

## Background

Non-small cell lung cancer (NSCLC) is a diverse disease with numerous molecular subtypes [1–4], and improved outcomes can be obtained through the matching of targeted therapies to their oncogenic drivers [3, 5–7]. To select appropriate targeted therapy, comprehensive molecular testing is recommended [7–10]. Next-generation sequencing (NGS)-panel testing offers broad molecular testing, providing comprehensive identification of oncogenic drivers for optimising targeted treatment selection [8, 11, 12]. While broad molecular testing is recommended, adoption of NGS varies globally, and one of the barriers to its implementation is the lack of an accepted standard for reporting results [13–15]. Although guidelines for pathology reporting exist [15–20], they do not specifically address the complexities of NGS data. Oncologists have expressed more confidence using single-gene tests, finding reports on multimarker tumour panel tests complicated, emphasising a need for improved NGS reporting and interpretation [14, 21, 22]. Standardisation and guidelines for reporting and interpreting NGS results are required for effective implementation of NGS testing in clinical practice. Here, we provide recommendations for reporting NGS-based panel testing results in NSCLC.

## Methods

Our recommendations were established through roundtable discussions between pathologists and oncologists, which were organised and supported by Merck Healthcare KGaA, Darmstadt, Germany (CrossRef Funder ID: 10.13039/100004755) and EMD Serono, Inc., Rockland, MA, USA, an affiliate of Merck KGaA (CrossRef Funder ID: 10.13039/100004755). All authors attended at least one of two meetings, in addition to medical writers who documented the discussions. A premeeting survey, developed with the guidance of Drs Malapelle and Rolfo, and completed by roundtable participants, gathered insights on current practices, key challenges, and areas for improvement for reporting NGS-based panel testing results based on the participants’ practical experience. The roundtable meetings discussed the needs of physicians, oncologists and pathologists, and aspects of NGS reporting that required improved standardisation. The recommendations based on these roundtable discussions are summarised below, and are intended to complement existing guidelines related to the use of NGS.

## Discussion/Observations and recommendations

### Pathophysiology of NSCLC and matching targeted treatments

NSCLC is a heterogeneous disease that can be broadly categorised by the presence or absence of oncogenic driver alterations [1–4]. Driver alterations are present in approximately 60% of lung adenocarcinoma cases, and define several molecular subtypes of NSCLC [3]. Targeted therapies matched to their oncogenic drivers are associated with improved survival and quality of life, and are recommended by clinical guidelines including European Society for Medical Oncology (ESMO), American Society of Clinical Oncology (ASCO), and NCCN Clinical Practice Guidelines In Oncology (NCCN Guidelines^®^) [3, 5–7]. The appropriate matching of patients with targeted therapies in clinical practice requires timely and comprehensive molecular testing, including genetic alterations with frequencies ≤1%, such as *RET*- or neurotrophic tropomyosin receptor kinase (*NTRK*)-fusions for which effective targeted therapy is available [8, 23, 24]. With the growing number of targeted therapies that are approved/under development, clinical guidelines recommend broad genomic testing approaches, such as tissue and/or liquid biopsy NGS-based panel testing [7, 9, 10]. Broad molecular profiling can also interrogate relevant co-alterations, such as resistance mutations for targeted therapies, or Kelch-like ECH-associated protein-1 (*KEAP1*) and serine/threonine kinase 11 (*STK11*) mutations, which are associated with resistance to immune checkpoint inhibitors [25].

### Assessment of molecular alterations in NSCLC using NGS

NGS-based panel testing enables many molecular alterations to be tested simultaneously, conferring several benefits over sequential single-gene approach, including tissue preservation, potential cost savings, and faster identification of patients with therapeutically targetable molecular alterations [8, 11, 12].

NGS involves high-throughput and comprehensive sequencing of deoxyribonucleic acid (DNA) or ribonucleic acid (RNA) [8, 12, 13, 26, 27]. DNA is more stable than RNA [28], facilitating convenient extraction from samples; however, DNA-based assays are less sensitive for gene fusions and alterations involving intronic regions (e.g. mesenchymal–epithelial transition factor exon 14 skipping) than RNA-based assays [26, 28]. Complementary NGS panels using DNA and RNA may therefore be required to cover all clinically relevant alterations with sufficient sensitivity [14, 27, 28].

Both DNA and RNA for NGS can be isolated from tumour specimens [21, 29, 30], and the majority of molecular testing has historically used tissue biopsies or cytological specimens as the ‘standard’ sample type [21, 29, 30]. However, circulating tumour DNA (ctDNA) in liquid biopsy can be used as an alternative source for NGS analysis [12, 29]. Moreover, circulating cell-free RNA (ccfRNA), including messenger RNA (mRNA) and micro RNA (miRNA), are also of interest as biomarkers for lung cancer, and liquid biopsy RNA sequencing is being developed [31, 32]. Liquid biopsy advantages include a minimally invasive collection procedure, repeatability, and, although so far not standard procedure, better evaluation of tumour heterogeneity and clonal evolution, with the ease of longitudinal monitoring of molecular response to treatment [12, 29, 33]. Limitations of liquid biopsy can include its lower sensitivity, with ctDNA NGS missing approximately one fifth of actionable alterations compared with gold standard tissue biopsy genotyping [34, 35]. Furthermore, cell-free DNA from non-tumour sources, including clonal haematopoiesis, may lead to false-positive findings, including *KRAS* and *TP53* mutations [36]. NGS sequencing can be performed using commercially available kits or with laboratory-developed tests, which can vary in how many genes are covered, and the algorithms used to identify alterations [8, 14, 26]. Liquid and tissue biopsy-based NGS analysis are complementary methods that enhance detection and sensitivity when used together [37].

Despite consensus in international guidelines on the importance of broad molecular profiling [3, 6, 7, 9], the adoption of NGS-based panel testing varies globally due to differing awareness levels, turnaround times, quality, access, costs, and reimbursement by health insurance [38, 39]. In the US, a large proportion of patients with NSCLC (64%) are not able to benefit from precision medicine due to clinical practice gaps, including preanalytical biomarker testing and post-analytical practice challenges [40]. One of the barriers to successful implementation of NGS in NSCLC is a lack of standards and guidelines specifically for the reporting and clinical annotation of its results [13–15]. Given the variety of methods available, and the volume and complexity of data generated by NGS, greater standardisation in reporting practices is necessary for oncologists to optimise patient care [13, 15, 21]. This need was highlighted in a 2020 survey which found that oncologists were more confident using single-gene tests than whole-genome or -exome sequencing to make clinical decisions, and the use of multimarker tumour panel tests was regarded as more complicated than single-gene tests, highlighting the need for improved reporting and interpretation of results obtained from multimarker panel tests [14, 21, 22].

Existing guidelines for pathology reporting have been summarised previously [15–20]; however, with the increased use of NGS-based panel testing and complexity of the data generated, specific guidelines for reporting NGS results are required. In this manuscript, we provide recommendations for best practice for the reporting of NGS-based panel testing results in NSCLC.

### NGS workflows

**Fig. 1:**
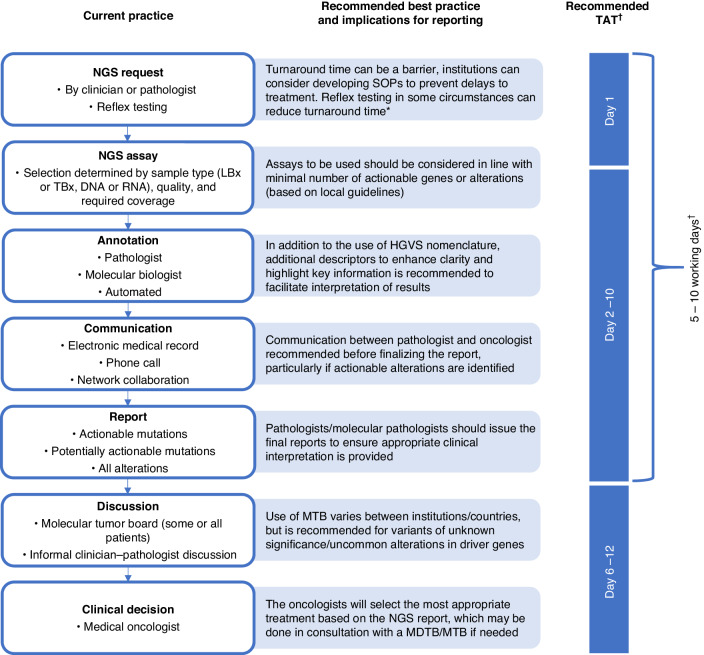
Typical NGS workflow. *Pathologist-directed reflex testing [43]. ^†^Provision of report within 5–10 working days from receipt of the sample recommended where possible [14, 21, 69], with the possibility of newer NGS platforms providing a faster TAT. DNA deoxyribonucleic acid, HGVS Human Genome Variation Society, LBx liquid biopsy, MDTB multidisciplinary tumour board, MTB molecular tumour board, NGS next-generation sequencing, RNA ribonucleic acid, SOP standard operating procedure, TAT turnaround time, TBx tissue biopsy.

There is inherent variability in NGS workflows between countries and between institutions in the same country [8, 14, 21, 38, 41]. A standardised workflow that can be implemented globally is not currently feasible; however, most workflows follow the broad seven steps summarised below [8, 12, 14, 31, 33, 42–49]. Our considerations for best practices and implications for reporting of NGS results, based on current evidence, are outlined in Fig. 1.

1. NGS request: The process for requesting NGS testing varies and may involve reimbursement considerations [14, 43]. Typically this is done by the oncologist, pathologist, a multidisciplinary tumour board (MDTB) or molecular tumour board (MTB), or by reflex testing (i.e. following histological diagnosis, particularly in the case of advanced disease, the NGS test is immediately ordered by the pathologist), as determined by local guidelines [8, 14, 43].
2. NGS assay: The patient’s clinical history, already known genetic alterations, previous tyrosine kinase inhibitor (TKI) therapy with specific searches for resistance alterations to targeted therapy, sample type, sample quality and quantity (tumour content), and required gene coverage are key factors in guiding the selection of appropriate assays (in addition to local availability of specific assays) [8, 15]. At this stage, histopathological review is essential to evaluate preanalytical variables relevant for test selection and interpretation, to inform optimal sample selection and, in case of rebiopsies, to confirm or update the diagnosis [33, 39, 50]. Details of the biopsy and key assay parameters or limitations including details regarding the gene coverage, limit of detection, and reference range, should be captured within the NGS report, to enable appropriate interpretation of the results [12–15, 21, 26–33, 38, 39]. In the report, a link could be included to the laboratory’s website containing information regarding the laboratory’s accreditation status, internal/external quality control, and mode of internal/external calibration of DNA/RNA measurements.
3. Variant annotation and interpretation: There are several genomic knowledge bases available, but they vary in content, format, and evidentiary standards for actionability [13–15]. Links in the report to the used databases might be helpful and may avoid duplication of efforts to identify actionable alterations by the report recipients. Laboratories should follow applicable local guidelines to determine the most appropriate database for use in their practice [14, 15]. Inclusion of Human Genome Variation Society (HGVS) nomenclature in the report is helpful for standardisation of molecular descriptions [13, 15, 44]. However, this may not be the most effective way of communicating key genetic data to clinical users and when using HGVS nomenclature, we recommend the use of additional descriptors to enhance clarity (e.g. scientific nomenclature or appropriate alternative nomenclature); for example, the *EGFR* mutation c.2573T > G (HGVS nomenclature) should also be described as p.Leu858Arg (L858R; scientific nomenclature). Additionally, key information should be highlighted in a simple, easy to read and understandable format that facilitates interpretation of results [13]. Further recommendations for variant annotation are discussed in detail later in this manuscript.
4. Communication: Where possible, early communication is recommended between the pathologist/molecular pathologist responsible for producing the NGS report and the oncologist, who will use the results, to discuss the results before the finalisation of the report [15]. The provision of clear questions from the oncologist to the pathologist/molecular pathologist, and discussion between the pathologist/molecular pathologist and the oncologist on findings and problems will help clarify what needs to be analysed and what information needs to be delivered in the report [15]. The report can then be tailored to address any questions and ensure all information is available in the version of record [15]. Although a discussion between the oncologist and pathologist is not required for every request, early communication when actionable alterations are detected can facilitate timely initiation of mutation-specific targeted treatment.
5. Generate report: Key areas for improving standardisation of reports are the overall order, with actionable alterations and a summary on the first page, clarity in variant annotation and interpretation, and ensuring sufficient information on assay parameters and sample quality is included to fully interpret results [12, 13, 15]. International Organisation for Standardisation (ISO) on reporting criteria for medical laboratories (ISO 15189) also recommend the inclusion of interpretation of results, and where relevant with explanatory or cautionary notes [14, 21]. To ensure accessibility of results, reports should always be integrated into electronic medical records [13, 15]. This may also facilitate linking of multiple biomarker tests to provide complete information [13, 15]. While graphic representations in reports may facilitate understanding, integrating these into medical records requires further optimisation in some systems [44]. Recommendations for the report structure/format are discussed in detail later in this manuscript.
6. Discussion: Depending on local guidelines and practices, a MDTB or MTB may discuss the NGS findings prior to a treatment decision [6, 12, 21, 39]. MDTBs/MTBs may facilitate decisions about the use of reflex testing in step 1 as part of standard operating procedures (SOPs) for liquid biopsy NGS analysis, and MDTBs/MTBs can provide valuable assistance to the oncologist in interpretation of the results, particularly where complex or rare variants are present [12, 14, 33]. The use of MTBs (including molecular pathologists, clinicians, geneticists, molecular biologists, and bioinformaticians) is recommended in the ESMO guidelines to improve the use of genetics-guided NSCLC care [6, 14]. Clinical context may also be needed when interpreting laboratory results, and the use of MDTBs can play an important role in providing clinical context to complex genetic information, which may optimise individual patient’s clinical management [21]. MDTBs are usually sufficient for typical mutations and first diagnosis cases, while MTBs may be more relevant for complex NSCLC cases (e.g. resistance to long-term targeted TKIs, and non-standard treatment options). Where appropriate, the use of virtual MDTBs/MTBs could be considered [21].
7. Clinical decision: Based on the findings of the NGS report, and discussion with an MDTB/MTB if required, the oncologist will select the most appropriate treatment or, if needed, look into clinical trial matching of the patient [8, 47]. To prevent delays to treatment initiation, it is recommended to implement NGS testing SOPs that dictate time-frames for each step.

### NGS reports

#### Overall structure and format

**Fig. 2:**
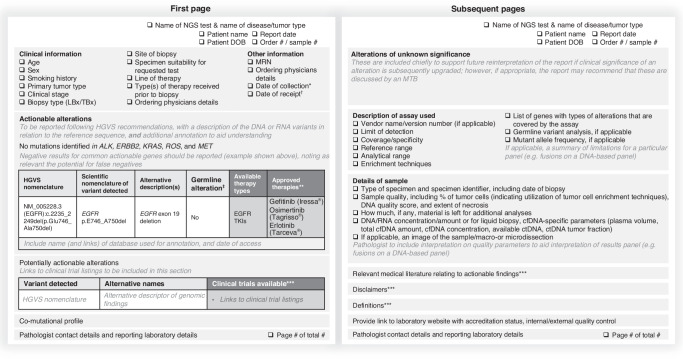
Diagram illustrating core elements and recommended structure of an NGS report. *Date of specimen collection. ^†^Date of laboratory receipt of specimen. ^‡^If germline variant analysis included in the assay. **Depending on country (should specify which relevant country/region the therapy is approved in). ***Optional. cfDNA circulating free DNA, ctDNA circulating tumour DNA, DNA deoxyribonucleic acid, DOB date of birth, EGFR epidermal growth factor receptor, HGVS Human Genome Variation Society, LBx liquid biopsy, MRN medical record number, MTB molecular tumour board, NGS next-generation sequencing, TBx tissue biopsy.

Given the volume of information generated by NGS testing, the results need to be structured such that information immediately relevant for clinical decision-making is readily available on the front page, while supporting and contextual information is contained on subsequent pages [13–16, 39]. A summary of the core elements that should be included, and which should be prioritised for inclusion on the first page is shown in Fig. 2.

In addition to the inclusion of unique patient identifiers, sample number, and dates relating to specimen collection and laboratory receipt, a section describing a minimal set of clinical information needed to interpret results should be included on the front page of the report [14–16, 20, 21, 51]. These parameters should include referral reason and clinical information such as age, biopsy type, site of biopsy, and where possible: smoking history, primary tumour type, clinical stage of the disease, previous molecular testing, line of therapy, and type(s) of therapy received prior to biopsy [14–16, 51]. While clinical information may be more readily available for in-house reports, for samples referred to external laboratories, it is useful if this additional demographic data is provided to the laboratory together with the ordering physician’s contact details, to allow for personal discussion on complex cases. This is particularly relevant when results are being reviewed in MDTBs/MTBs, to ensure NGS findings can be discussed in the clinical context of the patient. If other NGS/biomarker analyses have been done, results should be included in the report so that sequential results (e.g. with serial liquid biopsy) can be reviewed during the discussion session, for evaluating tumour evolution and most appropriate treatment(s) or sequence of treatments [8, 13, 16, 52].

A clear summary of any actionable, potentially actionable alterations, negative results for common actionable genes (including, as relevant, any caveats regarding the potential for false-negatives), and a list of relevant treatments, should be included on the first page, and annotated according to the ESMO Scale for Clinical Actionability of molecular Targets (ESCAT) or a comparable locally implemented scoring system to enhance the readibility [8, 14–16, 21, 52]. This summary with the relevant interpretation and advice should be prominent and easy to understand to minimise the risk of missed opportunities to match patients with appropriate therapies or relevant clinical trials [14–16, 20, 51, 52]. This is particularly important outside of academic centres where oncologists may be working across several tumour types, and be less familiar with implications of specific alterations in NSCLC. In addition to actionable driver alterations, the report may highlight potentially relevant co-mutations for targeted therapies or immunotherapy [25]. This section can support multidisciplinary discussion to clarify the clinical relevance of the co-mutational profile and so inform treatment decisions. Variants of unknown significance in any gene should also be captured to support future clinical decision-making in cases where the clinical significance of the variant is subsequently upgraded [53]. However, this should be separate from the actionable and potentially actionable alterations that are highlighted on the first page [13]. Interpretation of results may be complicated if many variants of unknown significance are identified with large NGS panels, and tier-based reporting and the use of ESCAT rankings can help to improve interpretation of the results [13, 21, 33, 52].

Additional sections to provide on subsequent pages include technical parameters of the assay used, sample quality (however, if the sample quality is poor, this should be highlighted on the first page of the report), relevant medical literature information where appropriate, and any required disclaimers [14–16, 20, 51].

#### Variant annotation and interpretation

To ensure a universal language, nomenclature used to describe alterations should follow HGVS recommendations, providing a description of variants at the DNA/RNA level in relation to the reference sequence [13, 18, 44, 54, 55], and should include the HGVS nomenclature version being used [19, 54]. However, scientific/alternative nomenclature should also be included, with alterations being described by commonly used and easily interpretable descriptions, which may be more familiar to oncologists [14, 15, 51]. For example, reporting of the *EGFR* mutation c.2369C > T (HGVS nomenclature) should be accompanied by a common descriptor such as p.Thr790Met or T790M. In addition, identifying insertions and deletions, or exon skipping mutations, from HGVS nomenclature requires knowledge of exon boundaries, and additional descriptors may be needed to provide clarity to the oncologist [14, 15, 51, 54, 55]. Inclusion of alternative or outdated gene names (e.g. *HER2*) should be considered to further aid oncologist recognition. The report may also annotate any germline variants identified, if assessed by the assay, considering issues around patient consent and preference regarding disclosure of germline variants [56]. Defining terminology, such as variant allele frequency and single nucleotide variants, in an appendix can also be considered to facilitate understanding of the findings of the report [51].

Inclusion of clinical significance of variants detected is strongly recommended; however, this relies on databases which will vary between regions and are often updated [13, 47]. The report should include a listing of the databases used (with the date of data retrieval), which may be achieved using text modules, and the incorporation of this information in the report would improve the transparency of how the data were obtained.

Actionable alterations are those which have approved targeted therapies [2, 3, 5, 6, 39]. Treatment guidelines adapted to regional regulatory approvals and drug availability should be used to determine which alterations are actionable [3, 6, 7]. Using either ESCAT, ESCAT-like scoring systems, or separate listings of actionable alterations according to the local approval status, improves interpretation and facilitates the use of the report by oncologists [21, 39, 52].

Potentially actionable alterations are those for which a matching targeted therapy may be available via clinical trials, and should be listed separately to actionable alterations [8, 15, 47]. Inclusion of local resources, such as regional study centre websites, is encouraged. Identifying locally available clinical trials for potentially actionable alterations may fall beyond the scope of work conducted by a laboratory but can also be discussed by MTBs [47]. Networking with larger academic centres or research associations may enable smaller diagnostic units to provide information about clinical trials. Of note, matching patients with potential clinical trials may provide an important route for drug access in regions where drug availability is a barrier.

#### Assay-specific parameters and limitations

In the report, information regarding assay specific parameters and limitations should include a list of genes (with information regarding the exon coverage) that are covered and the types of alterations (mutations, copy number gains, fusions), germline variant analysis, version of the kit used, manufacturer details and instrument types, limit of detection/sensitivity (lowest detection limit for copy number alterations of the assay, analytical and technical sensitivity of the assay), coverage/specificity (read depth and completeness, with reporting of any potential presence of contamination that may limit analysis), reference range, analytical range (gene panel size; and if needed in the case of particularly large panels, the details of the assessed genes can be provided in a supplementary section), and enrichment techniques [8, 12, 14–16, 20, 48, 51, 57].

A clear discussion of potential technical shortcomings of the employed assay should be included, e.g. false negative rates of DNA-based panels or amplicon-based techniques for fusions and rare alterations, or the inability to determine the expression of novel fusions based on DNA-based assays; such information will also assist in the planning of additional testing [14, 16, 51, 57, 58]. If applicable, a note on limitations for particular types of panels can also be included (e.g. the limitations for detecting fusions with DNA-based panels), and if additional tests are recommended (e.g. RNA-based NGS or immunohistochemistry (IHC) to assess the expression of novel fusions) [15, 16, 58]. In addition, the report can identify patients whose available results may suggest limited benefit from further testing, such as those with *KRAS* mutations [7].

Further awareness regarding which panels/analytical approaches are validated for clinical use, and how suitable different assays are for detecting relevant variants, would be valuable to aid interpretation of results [13].

#### Sample quality

Differences in preanalytical conditions impact the interpretation of NGS results and must therefore be captured within the report [14, 57]. To facilitate understanding, this section of the report should be highly structured with interpretation by the pathologist. Relevant information that should be documented in this section of the report includes: (i) type of specimen, specimen identifier, and date of biopsy [14, 15]; (ii) sample quality, including percentage of tumour cells (utilisation of any tumour cell enrichment techniques [macro- or microdissection] should be included), DNA quality score, and extent of necrosis [14, 33, 51, 57]; (iii) how much, if any, material remains for additional analyses; (iv) DNA/RNA concentration/amount; or for liquid biopsy, circulating free DNA (cfDNA)-specific parameters including plasma volume, total cfDNA amount, cfDNA concentration, amount of available ctDNA, and ctDNA tumour fraction, where feasible [14, 16]; (v) contact data of the responsible molecular pathologist who is able to help the clinician interpret/use the report. For liquid biopsies, pre-analytical variables could be included (e.g. date/time of blood draw, date/time of laboratory receipt for separation/extraction).

The sample quality section is essential for interpreting the strength of any findings. This section should be highly structured with a clear interpretation by the pathologist, with any limitations or cautionary comments clearly noted [14, 16, 21, 51]. For example, where the neoplastic cell content was below the required threshold, the report should indicate if biomarker-negative results should be regarded as inconclusive [57], while, for liquid biopsies with low tumour fraction, the elevated risk of false-negative and false-positive findings should be noted [59, 60]. In cases where no alterations are found, this information will be used to differentiate negative findings from a non-diagnostic report and inform the appropriate course of action, such as the need for complementary testing.

### Future directions

Future directions in this landscape may include: (i) the integration of complementary tests such as liquid and tissue biopsy data in the report to provide complete biomarker reporting (e.g. inclusion of programmed death-ligand 1 expression status from tissue biopsy assessed through immunohistochemistry, together with other druggable genetic alterations identified through plasma NGS); (ii) utilisation of serial NGS (plasma or tissue) for disease/longitudinal monitoring of: genetic alterations/biological changes in the disease over time including mutant allele frequency (MAF)/clonal fluctuations, response to treatment, and acquired resistance mechanisms; (iii) development of predictive markers to new targeted agents, or for immunotherapy – potential predictive markers in the liquid microenvironment; (iv) further integrating the impact of co-mutations on targeted therapies or immunotherapy; (v) following tumour resection in the early stage of the disease, potential assessment of minimal residual disease based on plasma tumour genetic material; (vi) utilisation of DNA methylation biomarkers to facilitate early detection of NSCLC, gain insights into epigenetic alterations, and predict prognosis; (vii) in addition to ctDNA, analysis of other plasma analytes that may provide valuable information about other biomarkers; (viii) gene cluster identification in NSCLC to identify patterns of resistance, predict treatment response, and guide alternative treatment strategies; (ix) using a graphic summary (similar to a heat map) to help clinicians understand in one glance what targetable drivers have been tested, if they are positive or negative, and which ones have not been evaluated yet; (x) using soft reports/electronic pathology reports with links to big data (e.g. large-scale genomic/genetic/proteomic databases), to facilitate diagnosis, staging and treatment; (xi) using ctDNA tumour fraction to guide confirmatory tissue testing; and (xii) investigating the potential of digital pathology and artificial intelligence to predict biomarkers from histopathology scans [30, 61–67]. A growing field of interest is the implementation of minimal residual disease (MRD) platforms, as well as the usage of cfDNA, as surrogate markers post-definitive therapy in early disease (surgery and/or radiation) [68]. Currently, there is limited data available in order to have a solid statement associated with this arena, however we believe that both cfDNA and methylation-based technologies will be part of our future practice in the near future.

## Conclusions

Overall, the oncologists’ need for clear and concise information to enable clinical decision-making requires the provision of all necessary information to accurately interpret NGS findings, which can be achieved in part through optimising how results are reported. The integration of the clinical picture into the interpretation section of the laboratory reports may further help to improve the management of NSCLC. We hope that the recommendations and considerations described in this manuscript, based on practical experience of NGS reporting in NSCLC, will facilitate further standardisation of NGS reporting in NSCLC to ultimately ensure that reports are fully interpretable and the most appropriate treatment options are selected based on robust molecular profiles in well-defined reports.
